# Landscape Vegetation Productivity Influences Population Dynamics of Key Pests in Small Avocado Farms in Kenya

**DOI:** 10.3390/insects11070424

**Published:** 2020-07-09

**Authors:** Nadia K. Toukem, Abdullahi A. Yusuf, Thomas Dubois, Elfatih M. Abdel-Rahman, Marian Salim Adan, Samira A. Mohamed

**Affiliations:** 1 International Centre of Insect Physiology and Ecology, Nairobi 00100, Kenya; tdubois@icipe.org (T.D.); eabdel-Rahman@icipe.org (E.M.A.-R.); madan@icipe.org (M.S.A.); sfaris@icipe.org (S.A.M.); 2 Department of Zoology and Entomology, University of Pretoria, Private Bag X20, 0028 Pretoria, South Africa; aayusuf@zoology.up.ac.za

**Keywords:** integrated pest management, ecosystem services, smallholder, vegetation productivity

## Abstract

Avocado (*Persea americana* Mill.) production contributes to the economic growth of East Africa. However, poor fruit quality caused by infestations of tephritid fruit flies (Tephritidae) and the false codling moth, *Thaumatotibia leucotreta* (Meyrick), hampers access to lucrative export markets. Remote sensing and spatial analysis are increasingly applied to crop pest studies to develop sustainable and cost-effective control strategies. In this study, we assessed pest abundance in Muranga, Kenya, across three vegetation productivity classes, *viz*., low, medium and high, which were estimated using the normalised difference vegetation index at a landscape scale. Population densities of the oriental fruit fly, *Bactrocera dorsalis* (Hendel) and *T. leucotreta* in avocado farms were estimated through specific baited traps and fruit rearing. The population density of *T. leucotreta* varied across the vegetation productivity classes throughout the study period, although not significantly. Meanwhile, *B. dorsalis* showed a clear trend of decrease over time and was significantly lower in high vegetation productivity class compared to low and medium classes. *Ceratitis cosyra* (Walker) was the most abundant pest reared from fruit with few associated parasitoids, *Pachycrepoideus* *vindemmiae* (Rondani) and *Toxeumorpha nigricola* (Ferriere).

## 1. Introduction

Agricultural landscapes contain a range of arthropod pests which influence crop productivity [1,2,3]. Pests benefit from spatial dominance of host plants and low activity of natural enemies, leading to population build-up and more damages to crops. The distribution of these resources varies across landscapes and time [4], and influence pest population dynamics [5,6]. The normalised difference vegetation index (NDVI) is a common remote sensed variable which derives from the combination of surface reflectance at near infrared and red wavelengths to indicate a particular property of vegetation [7]. Numerous studies have demonstrated the use of NDVI as a proxy of vegetation productivity at the field or landscape scale. Vegetation productivity is the result of cumulative effect of biotic, abiotic and anthropogenic factors. Therefore, NDVI as a measure of vegetation productivity, interacts with farm or landscape abiotic factors like rainfall, temperature and altitude. The literature shows a strong linear relationship between NDVI and rainfall as well as temperature in humid and temperate regions [8]. However, in warm and tropical regions which are characterised by distinct dry and rainy seasons, there might be a weak relationship between NDVI and rainfall or temperature [9,10,11,12]. The normalised difference vegetation index also indicates insect pest habitat conditions and temporal changes in the landscape structure, which also represents shifts in pest resources [13,14,15]. Several studies have pointed out that models with NDVI as an indicator of vegetation productivity predicted pest spatial distribution more accurately than models without [16,17,18]. Therefore, NDVI can be used as an indirect approach to measuring the abundance and distribution of animal species. For crop pests, this approach is a potential way of assessing high-risk infestation areas, while providing context-specific management decisions.

Avocado, *Persea americana* Mill. (Lauraceae), is one of the most important horticultural crops grown in Kenya for subsistence, local markets and exports [19,20]. Tephritid fruit flies and the false codling moth, *Thaumatotibia leucotreta* (Meyrick) (Lepidoptera: Tortricidae), are the most important pests of avocado in sub-Saharan Africa [21,22,23,24,25]. The oriental fruit fly, *Bactrocera dorsalis* (Hendel) (Diptera: Tephritidae), makes 90% of pest insects trapped in avocado farms in Kenya and Tanzania [25]. *Bactrocera dorsalis* is a polyphagous pest, attacking more than 300 species of wild and edible hosts [26]. Females oviposit in the fruit mesocarp, and larvae hatch and feed by tunneling inside the fruit. Due to its polyphagous nature coupled with a high dispersal ability and reproductive rate, it is considered one of the most devastating pests of horticultural crops [27]. *Bactrocera dorsalis* is rapidly spreading across the world and is now present in 75 countries in Africa, Asia, North America, South America and Oceania [28]. In Europe, *B. dorsalis* is absent except in Italy, where the pest was recorded in 2018 [29]. The presence of alternative crops, such as mango, *Mangifera indica* L. (Anacardiaceae) and guava, *Psidium guajava* L. (Myrtaceae), help maintain the *B. dorsalis* population throughout the year. Other tephritid species, such as *Ceratitis cosyra* (Walker), *Ceratitis rosa* Karsch and *Ceratitis capitata* (Wiedemann) (all Diptera: Tephritidae), also damage avocado fruits [23]. Contrary to *B. dorsalis*, the mango fruit fly, *C. cosyra*, is native to Africa and has a restricted host range that includes mango, guava and wild fruits [30]. *Ceratitis cosyra* females damage fruits like *B. dorsalis*. *Ceratitis cosyra* was competitively displaced from mango by the invasive *B. dorsalis* [31].

The false codling moth *T. leucotreta* is a polyphagous pest in sub-Saharan Africa, but it prefers *Citrus* sp., *Capsicum* sp. and *Solanum* sp. [32]. Recently, *T. leucotreta* has become a significant pest of avocado in East Africa [25]. In Kenya, *T. leucotreta* was reared from 86 wild and cultivated species belonging to 33 plant families [33]. The female oviposits on the fruit skin, and larvae penetrate and tunnel inside the fruit. In avocado, larvae exit fruits as 5 instar and pupate in the soil [34].

The production of avocado in Kenya has seen steady growth, reaching 194,279 tons in 2017, up from 93,639 tons in 2007 [35]. Direct damages of pest infestations result in massive yield reduction and poor fruit quality, whereas indirect damage leads to fruit rejection in international markets due to quarantine restrictions. Fruits including avocado, which are exported from Africa, are frequently rejected by the European markets to limit the invasion of *B. dorsalis* [36] and *T. leucotreta* [37]. Since 2007, quarantine restrictions on the export of Kenyan avocado to South Africa result in an annual loss of US $2 million [38].

This study was carried out to assess the influence of vegetation productivity on the distribution and abundance of key avocado pests: *B. dorsalis*, *C. cosyra* and *T. leucotreta*, as well as their associated parasitoids in small avocado farms in Muranga, Kenya. Here, we expected a relationship between NDVI and temperature, and rainfall. We hypothesised that vegetation productivity, measured using NDVI, influences avocado pest populations. The landscape of the study area was grouped into three classes based on the defined NDVI value thresholds. Specific baited traps were deployed on the farms throughout the season to monitor pest populations dynamics. In addition, fruits were reared to record other pest species and parasitoids. This study sought to better understand the spatial occurrence of pests, which will be used in the development of sustainable and cost-effective pest management practices that will increase farmers’ productivity.

## 2. Materials and Methods

### 2.1. Study Site and Characterization of the Landscape

**Figure 1:**
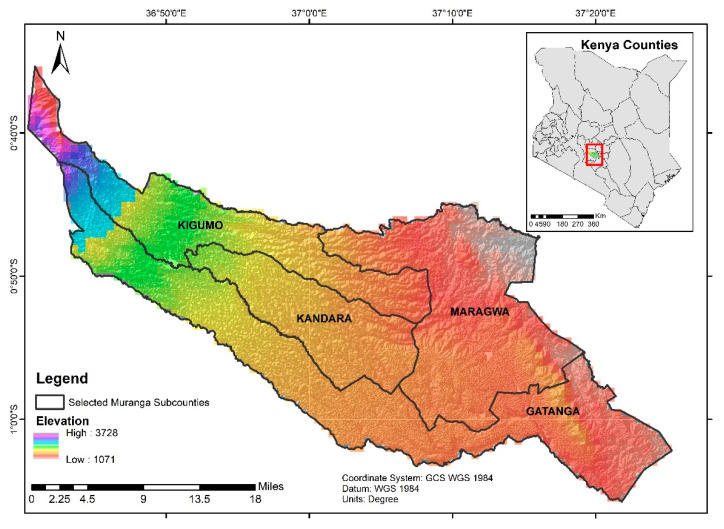
Location of the study area, Muranga county, Kenya. The background is the altitude range calculated from the Advanced Spaceborne Thermal Emission Reflection (ASTER) Global Digital Elevation Model (GDEM).

The study was carried out in Muranga county (0°43′ 0.01″ N, 37°08′60.00″ E) in the Central Region of Kenya, one of the major avocado producing areas [39]. The altitude ranges from 1071 m to 3353 m above sea level (asl) along the slopes of Aberdare forest in the west of the county (Figure 1). Muranga is well known for large plantations of tea, *Camellia sinensis* (L.) Kuntze (Theaceae), in the north, and commercial plantations of eucalyptus, *Eucalyptus* sp. (Myrtaceae), pineapple, *Ananas comosus* (L.) (Bromeliaceae) and avocado in the south. Landscape characterization was based on the vegetation productivity estimated by NDVI [40,41]. Freely available multi-date Sentinel-2 (S2) satellite data of 10 m spatial resolution were used to create a composite image with images of wet (March to May) and dry (December to February) seasons in the year 2019. We used multi-date imagery to account for the expected temporal variabilities in the vegetation productivity during the both wet and dry seasons in our study area. Satellite images were processed and analysed using the Google Earth Engine platform [42]. NDVI was computed as the ratio of the differences between the reflectance at near-infrared (NIR) and red (R) bands and their summation [(NIR − R)/(NIR + R)] [43]. The *K*-means unsupervised clustering method was used to differentiate three classes of NDVI, low, medium and high. *K*-means is a machine learning algorithm that clusters the dataset into *K* number of clusters, whereby each cluster contains data points close to the cluster’s mean value [44]. To cluster the multi-date NDVI imagery, we generated 100 random points using the ‘create random points’ tool in QGIS version 3.10.2 (https://qgis.org/downloads/) [45]. The NDVI values at these 100 points were extracted and put into the Past 3 tool [46] to employ the *K*-means method. The NDVI range values of each of three classes (i.e., low, medium and high) were used as thresholds to re-classify the multi-date NDVI imagery. The NDVI threshold for the low class was −0.425–0.368, while for the medium and high classes, thresholds were 0.368–0.611 and 0.611–0.864, respectively.

Furthermore, we acquired long term (2010–2018) monthly rainfall and temperature datasets from the Worldclim2 data platform (https://www.worldclim.org/data/monthlywth.html) [47]. Altitude was calculated from the Advanced Spaceborne Thermal Emission Reflection (ASTER) Global Digital Elevation Model (GDEM) version 3 (ASTGTM) (https://earthdata.nasa.gov/learn/articles/new-aster-gdem) [48]. These datasets were resampled to 10 m spatial resolution, and their values at the 100 randomly generated points for the NDVI were extracted. To select covariates that are independent of one another [49], we assessed the relationships between NDVI and rainfall, temperature and altitude using the Pearson correlation. A threshold of correlation coefficient of |r| = 0.70 was set to select the least correlated variable with NDVI. We found that both rainfall (r = 0.88; *P* < 0.0001) and temperature (r = −0.76; *P* < 0.0001) were strongly correlated with NDVI, while altitude was weakly correlated with NDVI (r = 0.46; *P* = 0.002). Therefore, we tested the effect of vegetation productivity and altitude on pest catches.

**Figure 2:**
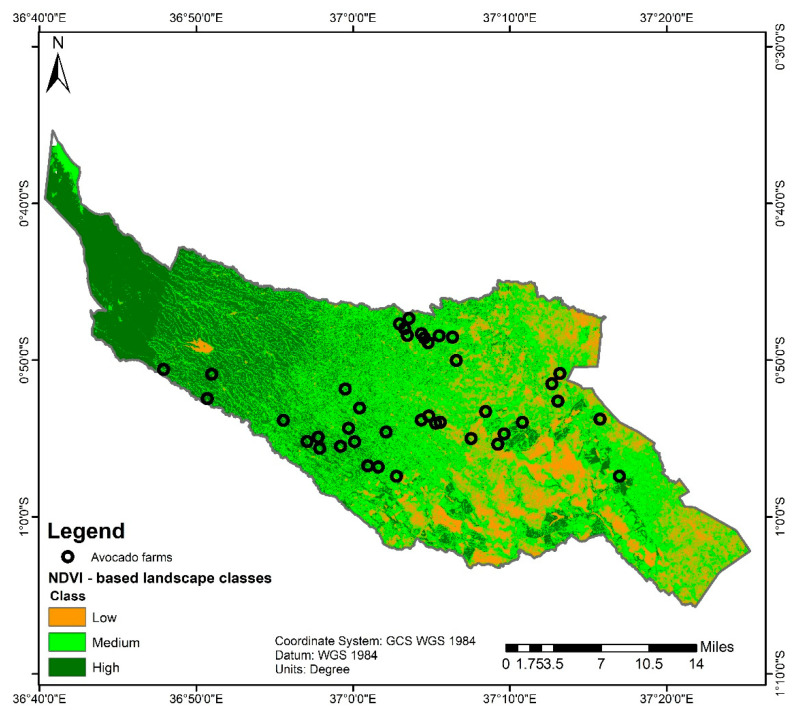
Distribution of avocado farms in Muranga County, Kenya across three normalised difference vegetation index (NDVI)- based landscape classification.

Small avocado farms (<0.4 ha) were selected in different villages to maximise the representativity of growers in Muranga. Within each vegetation productivity class, four administrative wards were targeted. Four farms located each in different village, between 0.5 to 9 km apart, were selected within each ward. Farms with a minimum of 15 trees of less than 30 years of age were selected. The willingness of the farmer to collaborate (security of traps, non-application of insecticide) during the study was also required. The farms were not sprayed with insecticides during the entire period of the study. However, a minimum of two farms was selected in some wards where we had a few potential avocado farms. Thirteen, twenty and seven farms were selected in high, medium and low vegetation productivity classes, respectively (Figure 2).

Observations were done in and 20 m around the selected farms to record the type of crops. Across all farms, avocado trees were grown in mixed cropping systems with staple food crops (maize, *Zea mays* L. (Poaceae); beans, *Phaseolus vulgaris* L. (Fabaceae); potatoes, *Solanum tuberosum* L. (Solanaceae)), fruit trees (mango, *Mangifera indica* L. (Anacardiaceae); macadamia, *Macadamia integrifolia* L. (Proteaceae)), coffee *Coffea arabica* L. (Rubiaceae) and/or tea. Mango trees were largely represented in low and medium vegetation productivity classes, while tea and coffee dominated in the high class. ‘Hass’ was the dominant avocado variety in the farms, whereas ‘Fuerte’, ‘Pinkerton’ and traditional varieties were under-represented.

### 2.2. Trapping of the Oriental Fruit Fly and False Codling Moth

Populations of *B. dorsalis* and *T. leucotreta* were monitored with traps baited with their respective lures between the fruiting period (end of February 2019) and the beginning of the flower bud stage (August 2019). Fruits were available in the fields throughout the study period because of the tendency of the avocado crop to bear fruits year-round. Lynfield traps (*icipe*, Nairobi, Kenya) baited with the para-pheromone methyl-eugenol (ME) (River Bioscience, Addo, South Africa) were used for *B. dorsalis* monitoring. The population of *T. leucotreta* was monitored with white delta-shaped traps baited with the sex-pheromone dispenser Crytrack [ (E)-8- and (Z)-8-dodecenyl acetate] (Kenya Biologics, Nairobi, Kenya). Two traps per farm, one for each species, were set 20 m apart along a 100 m transect. The transect ran in the middle of the farm, 30 m away from the edges and oriented from north to south. At each trap location along the transect, traps were hung at the east side of the tree, inside the upper 1/2 of the canopy and 2/3 outwards from the tree trunk. Insect samples were collected from traps every two weeks. The choice of this frequency was made to record population densities of a minimum of one generation, as the mean generation time is approximately 31 days and 48 days for *B. dorsalis* [50] and *T. leucotreta* [51], respectively. Insects from the Lynfield trap were preserved in 70% ethanol. The white glued paper from the delta-shaped trap was wrapped inside a polythene sheet. Traps were switched in the transect monthly to limit bias due to trap position. *Thaumatotibia leucotreta* pheromone dispensers were renewed monthly while the ME coats were replaced after every six weeks. In the laboratory, insects were identified, counted and recorded. Keys described by De Meyer (1998) [52], Drew & Romig (2016) [53] and Gilligan & Epstein (2014) [54] were used for the identification of fruit flies and moths.

### 2.3. Rearing of Pests and Parasitoids from Fruit

Avocado fruits were sampled monthly during the harvesting peak from February to May 2019. Eight pieces of fruit each from trees and the surrounding ground were collected per farm for further incubation in the laboratory. Four trees spaced at 20 m apart and located along the transect which had traps were targeted for fruit sampling. Fruits were randomly collected on the ground in a 2 m radius from the tree. Simultaneously, fruits with damage symptoms such as whitish perseitol exudates, holes and black spots were picked from the same tree. Fallen fruits were easier to find in some farms than in others because farmers usually feed livestock with fruits. In instances where the required number of fruits (i.e., 8) were not found on the ground, only 2 to 4 pieces were collected. Tree and ground-collected fruits were kept separately inside 20 L × 12 W × 40 H cm brown paper bags and transported to *icipe’s* Duduville campus, Nairobi (1°13′18.96″ S, 36°53′47.96″ E) for processing and incubation. While in the laboratory, fruits were counted, weighed, placed into 10 L plastic containers (Kenpoly, Nairobi, Kenya) on a thin layer (0.5 cm) of sterilised sand and incubated in the laboratory (24 °C ± 0.4 °C, 12D:12L, 70% RH). The lids of the containers were perforated at the middle and sealed with a fine mesh (1 mm) to provide ventilation. A total of 1030 pieces of fruit were collected during the study. Fruits were inspected every other day, and emerging pupae were transferred to Petri dishes. Emerged tephritid fruit flies, *T. leucotreta* and parasitoids were kept alive in the Petri dishes for four days to allow a full development of morphological features. Specimens were preserved in 90% ethanol for further identification.

Chalcidoid parasitoids were identified by Gerard Delvare, CIRAD, France (Chalcididae); John Noyes, The Natural History Museum, United Kingdom (Encyrtidae); Mircea-Dan Mitroiu, Alexandru Ioan Cuza University Iasi, Romania (Pteromalidae). The Nyssonidae specimen was identified by Robert Copeland using Bohart & Menke (1976) [55].

### 2.4. Data Analyses

Catches of *B. dorsalis* and *T. leucotreta* were expressed each as the daily capture per trap using the formula: total number of the insect caught/(number of serviced traps × number of days traps) [56]. The daily capture was averaged for each collection within each vegetation productivity class. Linear mixed models were performed to analyse daily catches of *B. dorsalis* and *T. leucotreta* as a function of landscape vegetation productivity and altitude. Collection time and farm were included as random effects. Full models were built with both random effects. Exceptionally for *T. leucotreta*, a correlation structure was added in the models to account for the spatial autocorrelation detected in *T. leucotreta* catches (Mantel’s test: r= −0.05; *P* = 0.99). Simplified models were defined from the full model by sequentially dropping the random effects and/or the correlation structure. The Restricted Maximum Likelihood approach was used to estimate model parameters and the best-fitting model was selected based on the lowest Akaike Information Criterion (AIC) value [57]. Model validation was done visually by assessing the homogeneity of residuals. Plots of residuals against fitted values were constructed for this purpose. The significance of the random effects was tested using the likelihood ratio test. The pair-wise comparison was implemented using Tukey’s test. Pests recovered from fruit incubation were used to calculate the number of emerged individuals per kg of fruit as a proxy of the infestation index [58]. The infestation index was estimated separately for ground-collected and tree-collected fruit. Data on the infestation index were analysed using the Kruskal-Wallis test to compare the median between vegetation productivity classes, followed by the Dunn’s test for posthoc analyses where *P* values were adjusted with the Benjamini-Hochberg method. Statistical analyses were performed in R version 3.6.3 [59], using the packages nlme [60], emmeans [61] and multcompView [62]. Significance was appreciated at α = 0.05.

## 3. Results

### 3.1. Farm Characteristics and Overview of Pests

Avocado farms in low and medium vegetation productivity classes were located between 1329 m and 1689 m asl, while farms in the high class were located between 1563 m and 2020 m asl. The most abundant fruit fly caught in ME-baited trap was *B. dorsalis* (99.6%). Few other fruit flies such as *C. cosyra, Ceratitis* (*Pardalapsis*) *cuthbertsoni* (Munro), *Ceratitis* (*Pardalapsis*) *ditissima* (Munro), *Perilampsis pulchella* (Austen) and *Ceratitis* (*Pterandrus*) *fasciventris* (Bezzi) were also caught. The false codling moth *T. leucotreta* was the most abundant moth species (99.8%) caught in the delta trap followed by the fall armyworm *Spodoptera frugiperda* (Smith) (0.2%).

### 3.2. Population Dynamics of Bactrocera dorsalis and Thaumatotibia leucotreta across Vegetation Productivity Classes

**Figure 3:**
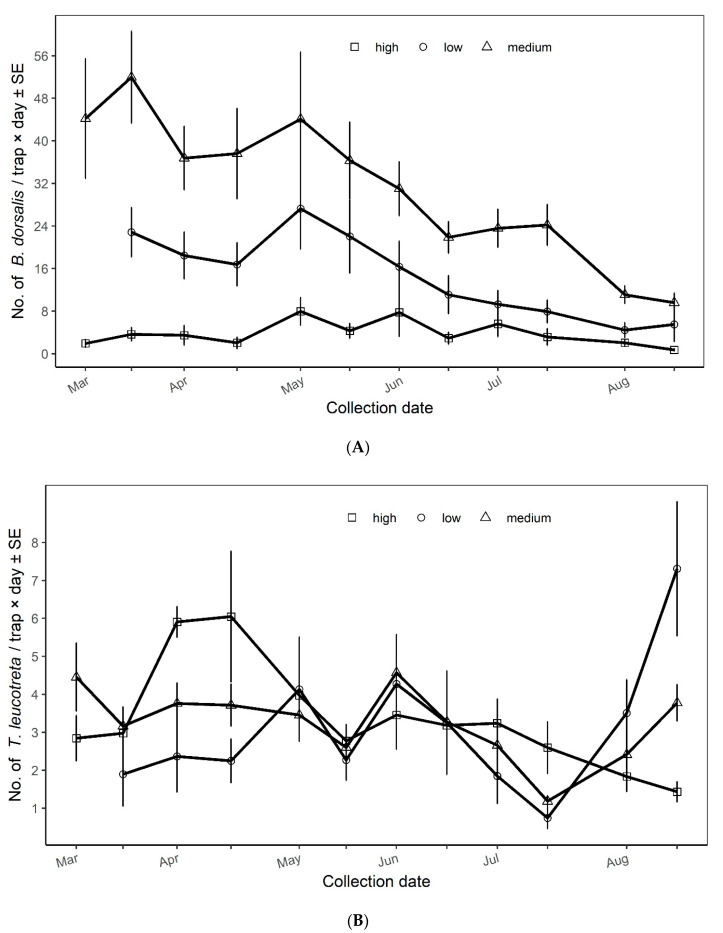
Population dynamics of (**A**) the oriental fruit fly *Bactrocera dorsalis* and (**B**) the false codling moth *Thaumatotibia leucotreta* in small avocado farms across vegetation productivity classes.

The population density of *B. dorsalis* varied between vegetation productivity classes throughout the season. In the low vegetation productivity class, there were two peaks of *B. dorsalis* catches, respectively in March and May 2019 (up to 25 flies/trap×day). After May 2019, catches of *B. dorsalis* started declining until the end of the study in August 2019 with 5 flies/trap×day (Figure 3A). A similar trend was observed in medium vegetation productivity class where the two peaks occurred also in March (52 flies/trap×day) and May 2019 (44 flies/trap×day). A gradual decrease of *B. dorsalis* was observed after May 2019 until the end of the study in August 2019 with daily captures of <16 flies/trap×day. Catches of *B. dorsalis* in high vegetation productivity class were relatively constant (<8 flies/trap×day) throughout the study. Slight increases of 6–7 flies/trap×day were recorded in mid-May and mid-June 2019 (Figure 3A).

The best-fitted model which explained *B. dorsalis* variation between vegetation productivity classes had both farm and collection time as random effects (AIC = 3584.50). Analysis indicated that the farm (χ^2^ = 153.25; df = 1,5; *P* < 0.0001) and collection time (χ^2^ = 63.93; df = 1,5; *P* < 0.0001) influence the variance of *B. dorsalis* catches. Landscape vegetation productivity significantly influenced *B. dorsalis* catches (F = 12.85; df = 2,37; *P* < 0.0001), being higher in medium vegetation productivity class, followed by low and high classes (Table 1).

Daily captures of *T. leucotreta* remained more abundant in the high vegetation productivity class (up to 6 moths/trap×day in May 2019) than in the low and medium classes, except in June and August 2019, during which the captures were more abundant in low and medium vegetation productivity classes than high class (Figure 3B). Trends in catches of *T. leucotreta* were similar in all vegetation productivity classes, except in May 2019 where daily captures increased in low vegetation productivity class while they decreased in high and medium classes. Also, in August 2019, daily captures decreased in high vegetation productivity class, while increasing in low and medium classes.

The correlation structure in *T. leucotreta* analysis did not improve the fit of the model (AIC = 2006.99). The best fitted model for *T. leucotreta* included both farm and collection time as random effects (AIC = 1979.99). The variation among farms (χ^2^ = 153.25; df = 1,5; *P* < 0.0001) and collection time (χ^2^ = 153.25; df = 1,5; *P* < 0.0001) explained the variance of *T. leucotreta* catches. However, there was not a significant effect of landscape vegetation productivity (F = 0.029; df = 2,37; *P* = 0.97) on the daily capture of *T. leucotreta* (Table 1). The mean daily capture of *T. leucotreta* slightly increased with the landscape greenness, being higher in high, followed by medium and low vegetation productivity classes (Table 1).

### 3.3. Distribution of Bactrocera dorsalis and Thaumatotibia leucotreta along the Altitude

Analysis showed that altitude strongly influenced *B. dorsalis* catches (F = 14.17; df = 1,38; *P* < 0.001). The daily capture of the pest decreased by 0.05 for every increase unit in altitude. In contrast, *T. leucotreta* did not vary with altitude (F = 0.011; df = 1,38; *P* = 0.91).

### 3.4. Emerged Pests and Parasitoids during Fruit Incubation

The mean mass per fruit varied between 150 g and 190 g. Fruit flies, *T. leucotreta* and parasitoids were recorded during fruit incubation, even though 7% of pupae failed to hatch. *Ceratitis cosyra* was the most abundant species (97.7%) recovered from fruits, followed by *B. dorsalis* (1.6%) and *T. leucotreta* (0.3%). The analyses focused only on the infestation index for *C. cosyra* because of its high prevalence. The *C. cosyra* infestation index of ground-collected fruit was significantly different across vegetation productivity classes (χ^2^ = 6.29; df = 2; *P* = 0.043) (Table 2). Fallen fruit was approximately three times more infested in low vegetation productivity class compared to high class. The infestation index of ground-collected fruit in areas of medium vegetation productivity (8.84 ± 1.69) was not significantly different from low (12.50 ± 3.10) and high (3.94 ± 1.31) ones. The infestation index of tree-collected fruit was negligible (between 0.14 ± 0.14 and 0.76 ± 0.75 flies/kg fruits) and not statistically different by vegetation productivity class (χ^2^ = 1.89; df = 2; *P* = 0.38).

**Figure 4:**
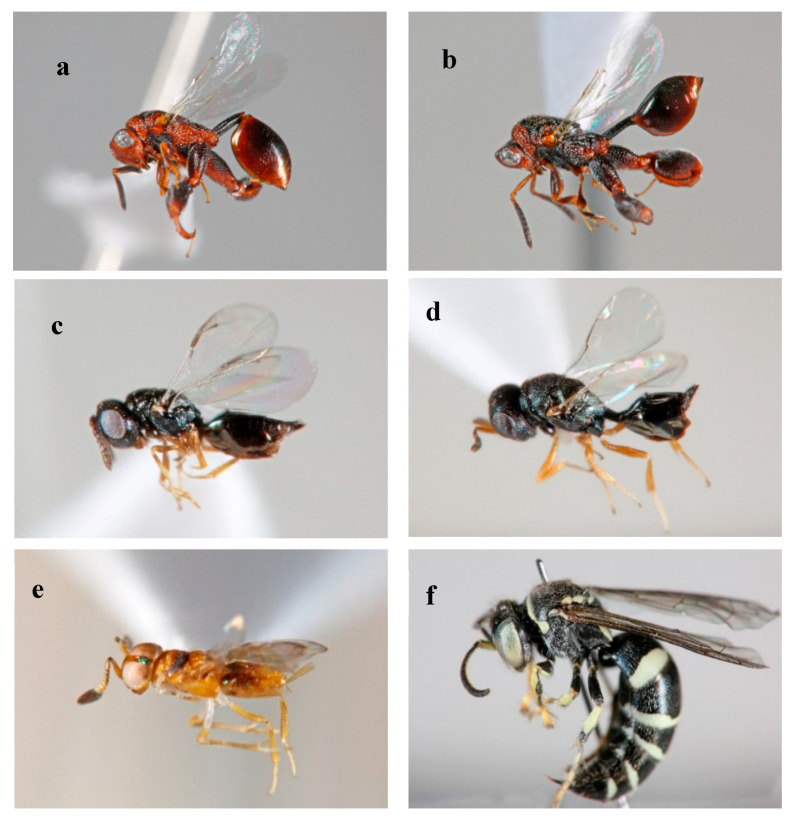
Parasitoids reared from avocado fruits (**a**,**b**) *Epitranus* sp. nov.; (**c**) *Toxeumorpha nigricola*; (**d**) *Pachycrepoideus vindemmiae*; (**e**) *Cheiloneurus sp*; and (**f**) *Bembecinus* sp.

Only 11 chalcidoid and one sphecoid wasps were recovered during fruit incubation. They were identified as *Epitranus* sp. nov. (7 individuals), *Cheiloneurus* sp. (one individual), *Pachycrepoideus* probably *vindemmiae* (Rondani) (two individuals), *Toxeumorpha* probably *nigricola* (Ferriere) (one individual) and *Bembecinus* sp. (one individual) (Nyssonidae) (Figure 4). Among them, *Pachycrepoideus vindemmiae* (the first record in Kenya) and *T. nigricola* emerged from *C. cosyra* pupae. Host pupae of *Cheiloneurus* sp., *Epitranus* sp. nov and *Bembecinus* sp. were not identified. *Epitranus* sp. nov. was found only in the medium vegetation productivity class, while *Cheiloneurus* sp., *P. vindemmiae*, *T. nigricola* and *Bembecinus sp*. were recorded in high vegetation productivity class. No parasitoid was recorded in low vegetation productivity class. Only one individual *Cheiloneurus* sp. was reared from tree-collected fruits in the medium vegetation productivity class.

## 4. Discussions

The fruit fly *B. dorsalis* was caught in ME-baited traps in the avocado farms throughout the season. Landscape vegetation productivity strongly influenced *B. dorsalis* populations. *Bactrocera dorsalis* catches were more abundant in medium and low than high vegetation productivity class. This involves that low and medium vegetation productivity are high-risk infestations areas and should receive considerable management efforts. Although vegetation productivity is a general measurement and does not reflect specific resources for the pest, it may probably have interacted with the presence of alternative host plants and influenced the distribution of *B. dorsalis*. Indeed, there was a gradual decrease in *B. dorsalis* captures over time across the three vegetation productivity classes. This trend is likely related to the phenology of avocado and alternative host plants such as mango. For instance, the presence of fruiting mango trees in and around avocado farms in low and medium vegetation productivity classes, as observed during the field sampling, would have also contributed to sustaining *B. dorsalis* populations, as mango is a primary host of *B. dorsalis* [22,27]. Also, *B. dorsalis* catches decreased with increase in altitude. This study further supports the characterisation of *B. dorsalis* as a lowland pest [25,50]. *Bactrocera dorsalis* populations in avocado farms decrease at higher elevations in Kenya and Tanzania [25]. Therefore, landscape vegetation productivity alone would not have influenced *B. dorsalis* population in avocado farms, but also the altitude. Future studies should specifically look at the influence of the abundance, distribution and diversity of host plants.

Unlike *B. dorsalis*, we found no significant effect of vegetation productivity and altitude on *T. leucotreta* captures. However, *T. leucotreta* was higher in high than low and medium vegetation productivity classes early in the season. Daily captures of *T. leucotreta* in low and medium vegetation productivity classes showed an increase in July 2019, probably because of the presence of alternative hosts such as macadamia and coffee which would have supported *T. leucotreta* populations when avocado trees were flowering and had few fruits. The trapping of *T. leucotreta* for a longer period (at least two years) might have given a clearer and consistent temporal trend of its population density across the landscape vegetation productivity classes. The lack of altitude effect on *T. leucotreta* can be explained by the thermal tolerance of the pest. Populations of *T. leucotreta* are sustained well with the presence of host plants in low and highlands areas in South Africa [63] and East Africa [25]. This reinforces that population dynamics of this pest may be driven more by the availability of hosts than vegetation productivity. The spatio-temporal distribution of *T. leucotreta* host plants need to be further investigated.

Unexpectedly, the mango fruit fly *C. cosyra* was the most abundant species reared from avocado. A potential explanation to this could be the high preference of *B. dorsalis* for mango over avocado in our study sites. *B. dorsalis* also prefers mango over *Citrus* sp., cucurbits and papaya [22]. This preference leads to the competitive displacement of *C. cosyra* by *B. dorsalis* on mango [31]. In our study, *C. cosyra* could have been displaced by *B. dorsalis* on mango and sought refuges in avocado. This involves fruit fly management in avocado farms also targeting mango trees in the farms. In future studies more emphasis will be put on the monitoring of *C. cosyra* with terpinyl acetate baited traps. *Bactrocera dorsalis* was the dominant species reared from avocado in Taita Hills (Kenya), a region where mango is rare, thus making avocado the main host plant for *B. dorsalis* [25]. *Thaumatotibia leucotreta* rarely emerged from ground-collected fruits. Females lay eggs on the fruit skin, and larvae hatch and tunnel inside the fruit. Exceptionally on avocado, larvae exit the fruit and pupate in a silken cocoon in the soil [34]. During our samplings, we probably have picked fruit from which nearly all larvae had already exited and pupated in the soil.

*Bactrocera dorsalis*, *C. cosyra* and *T. leucotreta* rarely emerged from tree-collected fruit, although infestation symptoms were recorded. Hanging and unripe avocado fruits develop defence mechanisms such as encapsulation and antibiosis to prevent larval development of fruit flies and *T. leucotreta* [23,24]. Also, the hardness and thickness of the exocarp of avocado varieties such as ‘Hass’ (the dominant variety in our study) make it difficult for female fruit flies to lay eggs underneath the skin. However, studies added that avocado’s defence mechanisms are reduced when fruits are ripe or punctured; this potentially explains why fruit flies and *T. leucotreta* were more abundant in ground-collected fruits than tree-collected fruits. Fruit fly species *C. cuthbertsoni.*, *C. ditissima, P. pulchella* and *C. fasciventris* caught in ME Lynfield traps were not found during the incubation of fruits. Therefore, these species likely are not pests of avocado and could have inadvertently trapped and killed by the malathion insecticide contained in the para-pheromone.

In this study, five parasitoids species were reared from avocado, among which *P. vindemmiae* and *T. nigricola* emerged from *C. cosyra* pupae. *Pachycrepoideus vindemmiae* is an idiobiont ectoparasitoid used in Hawaii, South America and Benin for the biological control of drosophilids and tephritid fruit flies such as *Bactrocera cucurbitae* (Coquillet), *Bactrocera latifrons* (Hendel), *C. capitata* and *B. dorsalis* [26,64]. *Toxeumorpha nigricola* is commonly found in Africa parasitising pupae of leaf-mining Lepidoptera and Diptera such as *C. capitata* [65]. However, the parasitism rate was very low in this study, suggesting the low ability of these species to invade avocados. *Toxeumorpha nigricola* may have a lower preference for *C. cosyra* than other hosts. Moreover, *P. vindemmiae* is a facultative hyperparasitoid attacking parasitised fruit flies [64], making it difficult to use the biological control. Species of larvae and pupae from which other parasitoids emerged were not well identified in our study. However, chalcid wasps such as *Epitranus* sp. and encyrtids such as *Cheiloneurus* sp. are occasionally reared from flies and may have parasitised drosophilids, calliphorids or muscoid flies which emerged during fruit incubation [66,67]. *Bembecinus* sp. like most of the sphecoids are mud daubers and would have been collected in the ground at the larval or pupal stage during the incubation of fallen avocado fruits. Further studies will help shed light on the host utilisation and efficiency of these parasitoids.

## 5. Conclusions

Fruit flies *B. dorsalis* and *C. cosyra* and the false codling moth *T. leucotreta* damage avocado fruits in Kenya. The vegetation productivity determined by NDVI influences catches of *B. dorsalis* but not *T. leucotreta*. Similarly, *B. dorsalis* populations increase with a decrease in altitude, while *T. leucotreta* do not vary with altitude. Landscape vegetation productivity is also a predictor for *C. cosyra* infestation of avocado fruits. In this study, very few parasitoids emerged during fruit incubation, among which the pteromalids *T. nigricola* and *P. vindemmiae* parasitised *C. cosyra*.

## Figures and Tables

**Table 1:** Mean daily capture and standard error (± SE) of *Bactrocera dorsalis*, *Thaumatotibia leucotreta* across landscape vegetation productivity classes.

	*B. dorsalis*		*T. leucotreta*	
Vegetation Productivity Class	N	Mean ± SE	N	Mean ± SE
Low	75	14.82 ± 1.53 ab	74	3.11 ± 0.36
Medium	206	29.86 ± 1.96 a	214	3.20 ± 0.15
High	142	3.68 ± 0.58 b	151	3.26 ± 0.44

Means within the same column followed by the same letter are not significantly different, Tukey’s test, α = 0.05.

**Table 2:** Infestation index for *Ceratitis cosyra* of the ground and tree-collected fruits across landscape vegetation productivity classes.

	Mean Number of Adults/kg of Fruits (± SE)	
Vegetation Productivity Class	From Ground	From Tree
Low	12.50 ± 3.10 a	0.14 ± 0.14
Medium	8.84 ± 1.69 ab	0.32 ± 0.20
High	3.94 ± 1.31 b	0.76 ± 0.75

Means within the same column followed by the same letter are not significantly different, Dunn’s test, α = 0.05.
